# Cerebral venous congestion alters CNS homeostatic plasticity, evoking tinnitus-like behavior

**DOI:** 10.1186/s13578-024-01221-9

**Published:** 2024-04-09

**Authors:** Huimin Wei, Huimin Jiang, Yifan Zhou, Lu Liu, Wei Ma, Shanshan Ni, Chen Zhou, Xunming Ji

**Affiliations:** 1 Beijing Advanced Innovation Center for Big Data-Based Precision Medicine, School of Biological Science and Medical Engineering, Beihang University, No. 37 Xueyuan Road, Haidian District, Beijing, 100191 China; 2 Laboratory of Brain Disorders, Collaborative Innovation Center for Brain Disorders, Beijing Institute of Brain Disorders, Beijing Advanced Innovation Center for Big Data-based Precision Medicine, Ministry of Science and Technology, Capital Medical University, No.10 Xitoutiao, You An Men, Beijing, 100069 China; 3 Department of Neurology, Xuanwu Hospital, Capital Medical University, Beijing, 100053 China; 4 Department of Neurology, Wuqing Hospital of Traditional Chinese Medicine Affiliated to Tianjin University of Traditional Chinese Medicine, Tianjin, 301700 China; 5 Department of Neurosurgery, Xuanwu Hospital, Capital Medical University, Beijing, 100053 China

**Keywords:** Cerebral circulation, Cerebral venous congestion, Brain metabolic activity, CNS homeostatic plasticity, Tinnitus

## Abstract

**Background:**

Brain function and neuronal activity depend on a constant supply of blood from the cerebral circulation. The cerebral venous system (CVS) contains approximately 70% of the total cerebral blood volume; similar to the cerebral arterial system, the CVS plays a prominent role in the maintenance of central nervous system (CNS) homeostasis. Impaired venous autoregulation, which can appear in forms such as cerebral venous congestion, may lead to metabolic abnormalities in the brain, causing severe cerebral functional defects and even chronic tinnitus. However, the role of cerebral venous congestion in the progression of tinnitus is underrecognized, and its pathophysiology is still incompletely understood. This study elucidated the specific pathogenetic role of cerebral venous congestion in the onset and persistence of tinnitus and the possible neurophysiological mechanisms.

**Results:**

We found that a rat model of cerebral venous congestion exhibited tinnitus-like behavioral manifestations at 14 days postoperatively; from that point onward, they showed signs of persistent tinnitus without significant hearing impairment. Subsequent neuroimaging and neurochemical findings showed CNS homeostatic plasticity disturbance in rats with cerebral venous congestion, reflected in increased neural metabolic activity, ultrastructural synaptic changes, upregulated synaptic efficacy, reduced inhibitory synaptic transmission (due to GABA deficiency), and elevated expression of neuroplasticity-related proteins in central auditory and extra-auditory pathways.

**Conclusion:**

Collectively, our data suggest that alternations in CNS homeostatic plasticity may play a vital role in tinnitus pathology caused by cerebral venous congestion. These findings provide a new perspective on tinnitus related to cerebral venous congestion and may facilitate the development of precise interventions to interrupt its pathogenesis.

**Supplementary Information:**

The online version contains supplementary material available at 10.1186/s13578-024-01221-9.

## Introduction

The cerebral venous system (CVS) contains approximately 70% of the total cerebral blood volume and plays a critical role in the maintenance of central nervous system (CNS) homeostasis [1–4]. Similar to the cerebral arterial system, the CVS has vascular regulatory functions (especially neurovascular and neurometabolic coupling) to ensure adequate spatially and temporally targeted delivery of energy substrates in accordance with neuronal activity [4]. Impaired cerebral venous regulation, such as cerebral venous congestion, is an important contributor to the pathogenesis of blood-brain barrier (BBB) disruption [5], cerebral blood flow (CBF) dysregulation [4], altered brain metabolism [4], cerebral microhemorrhages [6], neuroinflammation [5, 7], and neurodegeneration [5, 8].

Recently, cerebral venous congestion has been recognized as a heterogeneous pathological process that is often accompanied by a series of nonspecific clinical manifestations, such as headache, visual impairment, and sleep disturbances [9, 10]. Of these, tinnitus, the most frequent complication, has a tremendous impact on patients’ psychological and physical health, leading to concentration difficulties, work hindrance, increased prevalence of depression and anxiety, and cognitive decline [11–13]. Markey et al. [14] reported that tinnitus is a representative symptom among cerebral venous congestion patients with idiopathic intracranial hypertension, with an incidence ranging from 52 to 60%. Our previous clinical study [10] found that long-term, continuous tinnitus was present in 60.5% of cerebral venous congestion patients. However, the role of cerebral venous congestion underlying the onset and progression of tinnitus remains incompletely understood.

In this study, we aimed to investigate whether cerebral venous congestion is associated with tinnitus-like behavior using a validated cerebral venous congestion rat model generated by bilateral extracranial and internal jugular vein ligation (JVL), as well as to elaborate possible neurophysiological mechanisms. We found that GABA deficiency occurred in rats with cerebral venous congestion, establishing a direct connection between metabolic abnormalities and neuroplasticity-related inhibitory mechanisms, which accelerate CNS homeostatic plasticity malfunction and thereby cause tinnitus-like behavior and persistent tinnitus. These findings provide a reference for understanding the pathological mechanism of cerebral venous congestion-evoked tinnitus and a new clue for the potential blockade of its development.

## Materials and methods

### Animals

Adult Sprague Dawley (SD) rats weighing 180–220 g were purchased from Vital River (Beijing, China). Animals were randomly divided into experimental and control groups. The rats were maintained in ventilated cages on a 12–12 light/dark cycle, temperature (23 °C ± 2 °C) standard laboratory conditions and given ad libitum access to food and water throughout the study at Capital Medical University. Rats were used for behavioral tests 0 day/7 day/14 day/1 month/3 month postoperatively. The animal data reporting followed the ARRIVE 2.0 guideline [15].

### JVL rat model

JVL model was generated as previously reported [16, 17]. Briefly, rats were deeply anesthetized with 4% isoflurane and subsequently maintained between 1.5 and 2% isoflurane during the surgical procedure using an isoflurane vaporizer (RWD, Shenzhen, China). Each rat was placed in a dorsally recumbent position under a dissection microscope, and the hair from the neck was removed. A cutaneous midline incision was made on the neck. Blunt dissection of the subcutaneous tissue was performed, and the dorsolateral salivary glands were separated. The internal jugular vein, located in the carotid sheath on both sides (above the internal carotid arteries next to the vagus nerve), was subsequently separated and exposed according to the published protocol of Auletta and co-workers [17], making an oblique incision cross one-third point on the orbital line near the ear, retracting the temporalis muscle dorsally and the exorbital lacrimal gland ventrally, exposing and ligating the retroglenoid vein, which is the extracranial termination of the transverse sinus in rodents. Surgical ligations were performed with 8 − 0 nonabsorbable surgical sutures. Sham rats received the same skin incision and operation without surgical ligation under isoflurane anesthesia. Once the surgery was completed, the wounds were closed using a 6 − 0 surgical suture with a simple continuous pattern. Rats were allowed to recover under the heat lamp, and antibiotic ointment was applied over the skin incision, following five consecutive days. All efforts were made to minimize the number of animals used and their suffering.

### Auditory brainstem responses (ABR)

Rats were deeply anesthetized with ketamine (100 mg/kg, i.p.) plus xylazine (10 mg/kg, i.p.). Needle electrodes were positioned subcutaneously beneath the pinna of the test ear (reference) and contralateral ear (ground) as well as at the vertex (active). Acoustic stimuli were elicited and evaluated by the Tucker Davis Technologies (TDT) System III hardware and SigGenRZ software (TDT, Alachua, FL, USA). Tone-burst stimuli were presented in 10 dB decrements from 90 dB sound pressure level (SPL) and the responses evoked were recorded at octave frequencies of 4, 8, 16, and 32 kHz. The ABR threshold was defined as the lowest stimulus intensity capable of producing repeatable (in at least two trials) ABR waves.

### Gap prepulse inhibition of acoustic startle (GPIAS)

The GPIAS was performed using the Acoustic Startle Reflex Starter Package for Rat (Med Associates, St. Albans, VT, USA), as described previously [18, 19]. In brief, GPIAS sessions consist of 30 gap and 30 non-gap trials. Conscious rats experienced testing with different band-pass-filtered sounds (1k Hz bandwidth centered at 4, 8, 12, and 16 kHz, respectively) at 65 dB SPL. Startle responses were elicited by a burst of white noise (20 ms, in an interval of 30–35 s randomly) at 110 dB SPL. The gap in the narrowband noise began 100 ms before the acoustic startle stimulus (50 ms, 5 ms rise/fall time). The interval between each startling noise was 30–35 s and each test took approximately 30 min.

### 18 F-fluorodeoxyglucose (18 F-FDG) microPET

The Department of Medical Imaging Research at the Central Laboratory of Capital Medical University provided the 18 F-FDG used in this study. Before the scan, all the rats were fasted (but with free access to water) for 12 h to maximize FDG uptake in the brain; the body weight of each rat and radioactivity of the syringe (before and after injection) were recorded for calibration of the effective dosage. After an uptake period of approximately 45 min following the intravenous 18 F-FDG injection through the caudal vein, the PET signals and hybrid computed tomography (CT) data were obtained with an Inveon MM micro-PET/CT scanner (Siemens Co., Ltd, Knoxville, TN, USA). By ordered subset expectation maximization 3-dimension/maximum a posterior probability (OSEM3D/MAP) algorithm and attenuation correction information derived from the CT, the 20-min list-mode PET data were binned into a single frame and reconstructed. The final voxel size and the matrix were 0.776 mm × 0.776 mm × 0.796 mm and 128 × 128 × 159, respectively. The SUV was calculated as: [body weight (g) × tissue activity concentration (kBq/cc) / effective injected radioactivity (kBq)]. Eventually, each regional SUVmean was measured for statistical analysis.

### Transmission electron microscopy (TEM)

Rats were anesthetized and intracardially perfused with 0.1 M phosphate-buffered solution (PBS) containing 4% paraformaldehyde (PFA) and 0.25% glutaraldehyde. The cerebral auditory-associated areas (amygdala [AMY], auditory cortex [ACx], medial geniculate body [MGB], inferior colliculus [IC], prefrontal cortex [PFC], hippocampus [HP], and cerebellum [CRB]) were delineated according to anatomical atlases [20]. The tissues were dissected, washed with 0.1 M PBS (pH 7.2), and then immersed in 2.5% glutaraldehyde for 2 h at 4 °C. The sample preparation of TEM was performed as previously described [21, 22]. Processed samples were observed under a JEM-2100 TEM (Philips). The number of synaptic vesicles, postsynaptic density (PSD) thickness, synaptic cleft width, and length of the synaptic active zone were measured and analyzed with ImageJ.

### GABA measurement

Rats were anesthetized and intracardially perfused with ice-cold PBS to remove blood. The brains were separated from the skull and rapidly cut into 2 mm thick slices. Samples were collected and homogenized in RIPA buffer (Solarbio), and the lysates were centrifuged at 10,000 g for 5 min to separate the supernatant. The concentration of total protein was determined using the Pierce™ BCA Protein Assay Kit (Thermo). The production of GABA was measured using an ELISA Kit for Gamma-Aminobutyric Acid (GABA; Cloud-Clone Corp) following the manufacturer’s protocols.

### RNA extraction and quantitative RT-PCR

Total RNA was extracted from each cerebral auditory-associated area using the RNAprep Pure Tissue kit (TIANGEN) and RNA concentration was measured by the NanoDrop spectrophotometer (Thermo). Then, cDNA was synthesized using FastKing gDNA Dispelling RT SuperMix (TIANGEN). Finally, the specific products of c-fos, early growth response gene-1 (EGR-1), and brain-derived neurotrophic factor (BDNF) were amplified by quantitative PCR using PerfectStart® Green qPCR SuperMix (TransGen Biotech) and QuantStudio™ 6 Flex Real-Time PCR System (Thermo). GAPDH was used as a normalization control. Details about the primers are summarized in Supplementary Table 1.

### Immunoblotting assay

Tissues of cerebral auditory-associated areas were lysed (RIPA buffer [Solarbio] with protease and phosphatase inhibitors [CST]) and equal amounts of supernatant proteins were separated by SDS-polyacrylamide electrophoresis (PAGE), and then transferred onto polyvinylidene fluoride (PVDF) membranes (Millipore). Next, PVDF membranes were incubated overnight at 4 °C with primary antibodies against c-fos (Proteintech, #66590-1-Ig, 1:1000) and β-III-tubulin (Biolegend, #802,001, 1:10000), followed by 1 h room temperature (RT) incubation with horseradish peroxidase-labeled secondary antibodies (ZSGB-BIO, 1:200). Labeled proteins were visualized using electrogenerated chemiluminescence (ECL) working solution (Millipore) and the density of each band was measured by ImageJ.

### Immunofluorescence microscopy

After being perfused through the heart with 4% (*w*/*v*) PFA in PBS (pH 7.4), brains were immediately removed, fixed overnight in 4% PFA, and immersed in 20% (*w*/*v*) and 30% (*w*/*v*) sucrose/PFA to dehydrate until they sunk to the bottom. Coronal brain sections (15 μm thick) were obtained using a freezing microtome (Leica) through the auditory areas according to the stereotaxic atlases [20]. For immunolabelling, slices were permeabilized with 0.3% Triton X-100/PBS for 30 min at RT, blocked with 10% goat serum/PBS for 90 min at RT, and then incubated with primary antibodies anti-c-fos (CST, #2250, 1:200) overnight at 4 °C. The next day, after washing in PBS, the slices were incubated with appropriate Alexa Fluor 488-conjugated secondary antibodies (1:200, ZSGB-BIO) for 90 min at RT. Subsequently, nuclei were stained with 4′,6-diamidino-2-phenylindole (DAPI; Sigma) for 10 min at RT. Images were acquired on a confocal laser scanning fluorescence microscope (ZEISS). Areas covered by antibody-fluorophores and their numbers were analyzed with ImageJ.

### Immunohistochemical analysis

The brain tissues were embedded in paraffin and sliced into 4-µm-thick coronal sections using a sliding microtome (Leica). After dewaxing, sections were boiled by microwave in retrieval solutions to expose antigens, followed by incubation in 3% (*v*/*v*) hydrogen peroxide (H2O2) for 30 min at RT. Then, sections were blocked with 10% (*v*/*v*) goat serum in 0.3% (*v*/*v*) Triton X-100/tris-buffered saline (TBS) for 90 min at RT and incubated with a mouse anti-c-fos antibody (Proteintech, #66590-1-Ig, 1:200) overnight at 4°C. The next day, after washing in TBS, the sections were reacted with biotinylated goat anti-mouse IgG (Vector Laboratories) for 90 min at RT. Subsequently, the slices were developed by VECTASTAIN Elite ABC-HRP kit (Vector Laboratories), and the immunohistological positive stainings were visualized by 3, 3’-diaminobenzidine (DAB) kit (Vector Laboratories) according to the manufacturer’s protocols. Finally, slices were counterstained with hematoxylin, dehydrated, and mounted. Images of brain tissue slices were captured with microscopic observation (Leica) and the quantification of c-fos positive nuclei were processed by ImageJ.

### Statistical analysis

Statistical analyses were performed with SPSS 19.0 (SPSS Inc., Chicago, IL) and GraphPad Prism 6 (GraphPad Software, La Jolla, CA). All the data are presented as the mean ± standard deviation (SD). Kolmogorov-Smirnov test was used to assess data distribution and Spearman’s rank correlation test was applied to analyze the correlation of different variables. The statistical significance of differences among more than two groups was tested by one-way ANOVA. Differences within the experimental groups were determined by two-way ANOVA and Student’s t test. All tests were 2-sided, and values of *P* < 0.05 were considered statistically significant.

## Results

### Rats with cerebral venous congestion exhibit tinnitus-like behavior

**Fig. 1:**
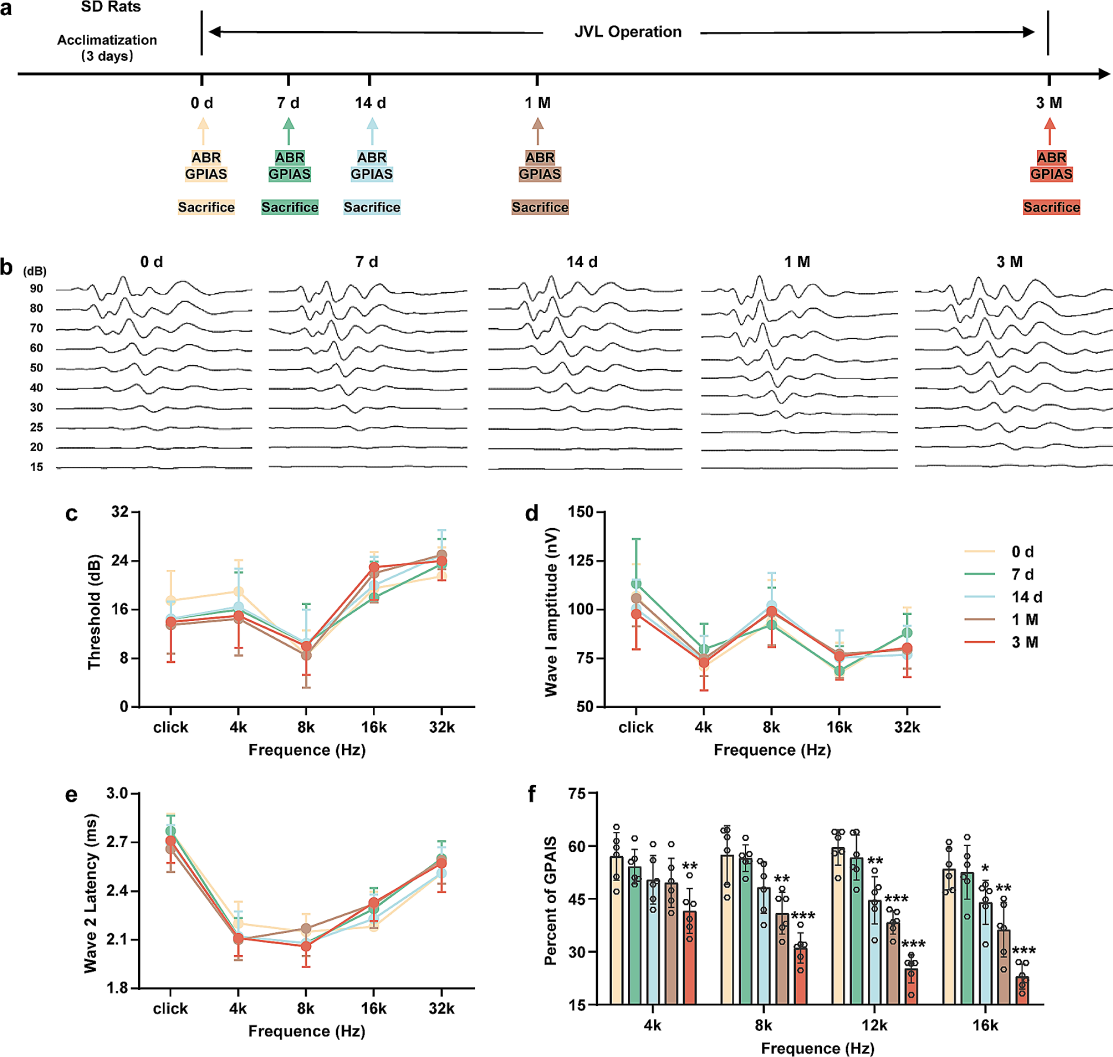
Effects of cerebral venous congestion on ABR thresholds and GPIAS values in rats. **a** Schematic diagram of the experimental design. Behavioral tests were performed at 0 d, 7 d, 14 d, 1 M, and 3 M after JVL. The color code (yellow, 0 d; green, 7 d; blue, 14 d; brown, 1 M; red, 3 M) is used for all results. **b-e** Representative ABR traces (**b**) as well as ABR thresholds (**c**), wave I amplitude (**d**), and wave II latency (**e**) (presented as line graphs) in response to clicks and to 4, 8, 16, and 32 kHz tones (*n* = 10). **f** Percent of GPIAS at 4, 8, 12, and 16 kHz (*n* = 6). All data are shown as the mean ± SD. Statistical significance was calculated using a two-tailed unpaired Student’s t test. Dots depict individual samples. ns, not significance; *p < 0.05; **p < 0.01; ***p < 0.001

After the successful establishment of a validated rat model of cerebral venous congestion by JVL [5, 6, 16, 17], behavioral tests were assessed before surgery as well as 7 days/14 days/1 month/3 months postoperatively (Fig. 1a). To investigate hearing function, we first measured the changes in the ABR threshold, the amplitude of ABR wave I, and the latency of ABR wave II (Fig. 1b-e). Across a broad frequency range (4–32 kHz), JVL rats showed elevated ABR thresholds and slightly prolonged wave II latencies, but there were no significant differences in the above hearing phenotypes among the five groups (Fig. 1c-e). Then, we performed GPIAS to explore whether cerebral venous congestion induced tinnitus-related behavior. Compared with rats from the 0 d group, rats from the JVL groups showed significant gap detection deficits at 4 kHz (3 M group, *P* < 0.01), 8 kHz (1 M group, *P* < 0.01; 3 M group, *P* < 0.001), 12 kHz (14 d group, *P* < 0.01; 1 M group, *P* < 0.001; 3 M group, *P* < 0.001), and 16 kHz (14 d group, *P* < 0.05; 1 M group, *P* < 0.01; 3 M group, *P* < 0.001) (Fig. 1f), indicating that these animals were experiencing tinnitus; such tinnitus-like behavior may appear 14 days after JVL. These findings demonstrate that rats with cerebral venous congestion have normal hearing function but exhibit tinnitus-like behavior, which is closely related to CNS metabolic activity.

### Cerebral venous congestion alters neural metabolic activity in auditory-associated areas

**Fig. 2:**
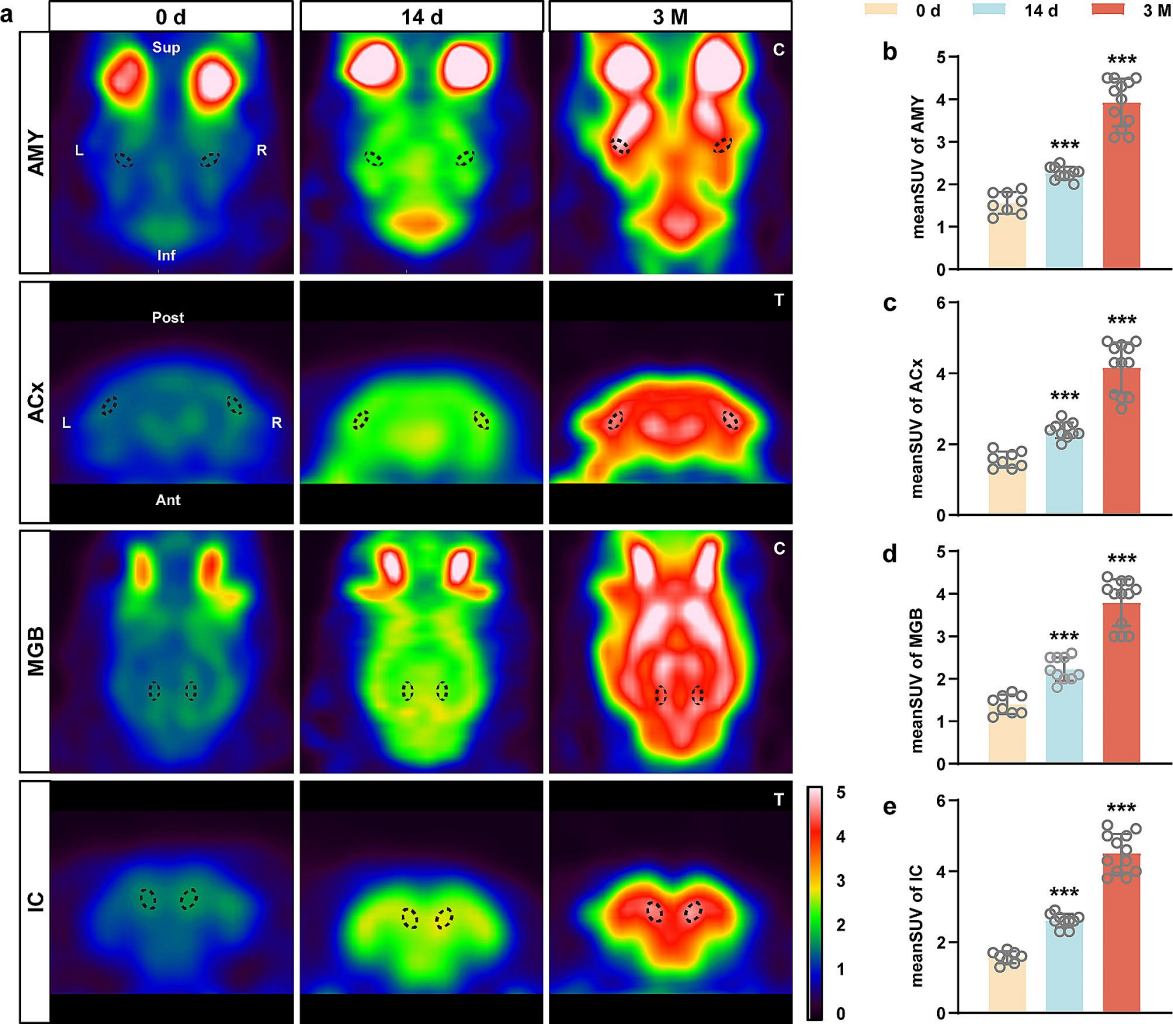
Neural metabolic activity was increased in the auditory-associated areas of rats with cerebral venous congestion. **a** Representative coronal or transaxial microPET images of JVL rats at 0 d/14 d/3 M postoperatively. Dotted areas identify AMY, ACx, MGB, and IC regions at the corresponding level. PET images are displayed according to an identical color scale; values ranging from 0 to 5 indicate the elevation of 18 F-FDG uptake. All rats were imaged in the prone position. Sup, superior; Inf, inferior; Post, posterior; Ant, anterior; L, left; R, right; C: coronal; T: transaxial. **b-e** Standard uptake value ratio of 18 F-FDG in the AMY (**b**), ACx (**c**), MGB (**d**), and IC (**e**). All data are presented as the mean ± SD (two-tailed unpaired Student’s t test) and are representative of at least five independent experiments. Dots depict individual samples. ***p < 0.001 compared to the 0 d group

PET imaging of 18 F-FDG is widely used to monitor cerebral metabolic patterns and identify the central auditory structures involved in tinnitus [23–25]. Thus, we investigated areas in the central auditory pathway (ACx, MGB, and IC) as well as nonauditory structures (PFC, AMY, HP, and CRB) to reveal the relationship between these brain regions and cerebral venous congestion-induced tinnitus. Corresponding transaxial, sagittal, or coronal microPET images of rats from the 0 d, 14 d, and 3 M groups are shown in Fig. 2 and Supplementary Fig. 1. Notably, visual comparisons of the images show evidence of enhanced FDG uptake in these brain regions at both 14 days and 3 months after JVL (Fig. 2a; Supplementary Fig. 1a).

The mean SUVs of the AMY (3.93 ± 0.56), ACx (4.16 ± 0.71), MGB (3.79 ± 0.55), IC (4.51 ± 0.55), PFC (4.08 ± 0.67), HP (4.15 ± 0.78), and CRB (3.67 ± 0.36) in the 3 M group were dramatically greater ( Fig. 2b-e; Supplementary Fig. 1b-d) than those in the other two groups; likewise, the 14 d group showed a statistically significant elevation in the mean SUVs of the AMY (2.26 ± 0.15), ACx (2.39 ± 0.22), MGB (2.23 ± 0.28), IC (2.61 ± 0.19), PFC (2.37 ± 0.34), HP (2.39 ± 0.31), and CRB (2.18 ± 0.30) relative to the 0 d group (Fig. 2b-e; Supplementary Fig. 1b-d), suggesting that the JVL rats were in a state of high CNS metabolic activity.

### Cerebral venous congestion induces ultrastructural alterations in synaptic endings

**Fig. 3:**
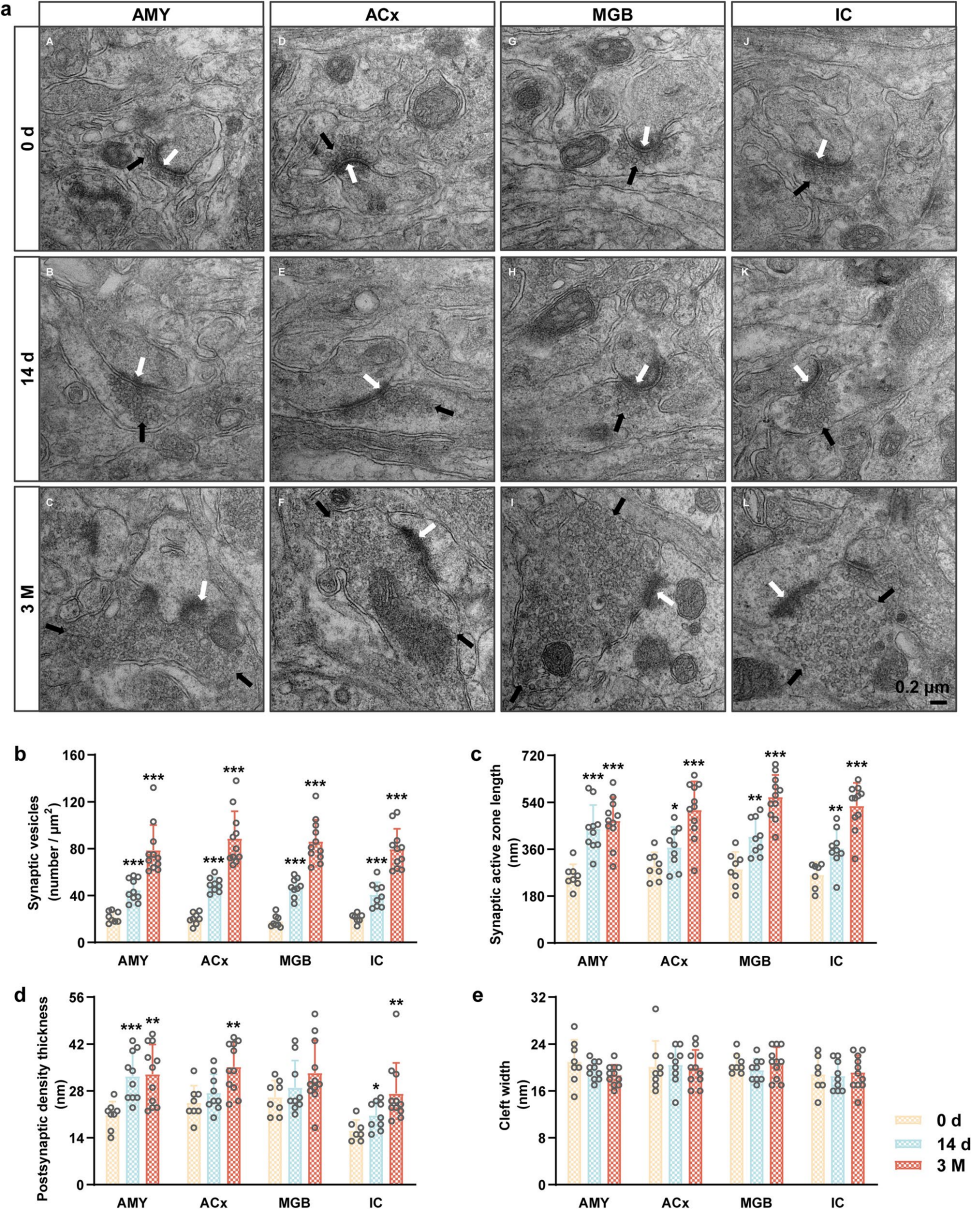
Cerebral venous congestion induced ultrastructural changes in synapses in the auditory-associated areas. **a** Representative electron micrograph of AMY, ACx, MGB, and IC sections in JVL rats at 0 d/14 d /3 M postoperatively. White arrowheads indicate PSD, and black arrowheads indicate presynaptic vesicles. Scale bar = 0.2 μm. **b-e** The number of synaptic vesicles (**b**), length of the synaptic active zone (**c**), PSD thickness (**d**), and synaptic cleft width (**e**) in the AMY, ACx, MGB, and IC. All data are presented as the mean ± SD (two-tailed unpaired Student’s t test) and are representative of at least five independent experiments. Dots depict individual samples. ns, not significance; *p < 0.05; **p < 0.01; ***p < 0.001

Hoping to determine whether the increased metabolic activity was accompanied by neuroplasticity-related changes, we assessed the synaptic ultrastructure (the structural basis of synaptic transmission and synaptic plasticity). Under TEM, the synaptic structure was legible, the outline was intact, and many synaptic vesicles were observed in the 0 d/14 d/3 M groups (Fig. 3a; Supplementary Fig. 2a). Quantitatively, the ultrastructure of the AMY/ACx/IC neurons contained more synaptic vesicles (Fig. 3b), longer synaptic active zones (Fig. 3c), and greater PSD thickness (Fig. 3d) in the 3 M group than in the 0 d group. Similarly, the ultrastructure of the AMY and IC neurons showed a significant increase in the number of synaptic vesicles (Fig. 3b) and synaptic active zone length (Fig. 3c) in the 14 d group compared to the 0 d group. More synaptic vesicles with longer synaptic active zones were present in the ACx and MGB neurons, but there were no significant changes in PSD thickness in the 14 d group (Fig. 3b-d). Also, there were no significant differences in synaptic cleft width between the three groups (Fig. 3e). Together, these observations suggest that both early and later stages of cerebral venous congestion can modulate the plasticity of synapses and neuronal circuits. Additionally, we discovered remarkable alterations in the synaptic ultrastructure of the PFC/HP/CRB neurons (Supplementary Fig. 2b-e).

### GABA links neuronal excitability signaling to cerebral venous congestion-evoked tinnitus

**Fig. 4:**
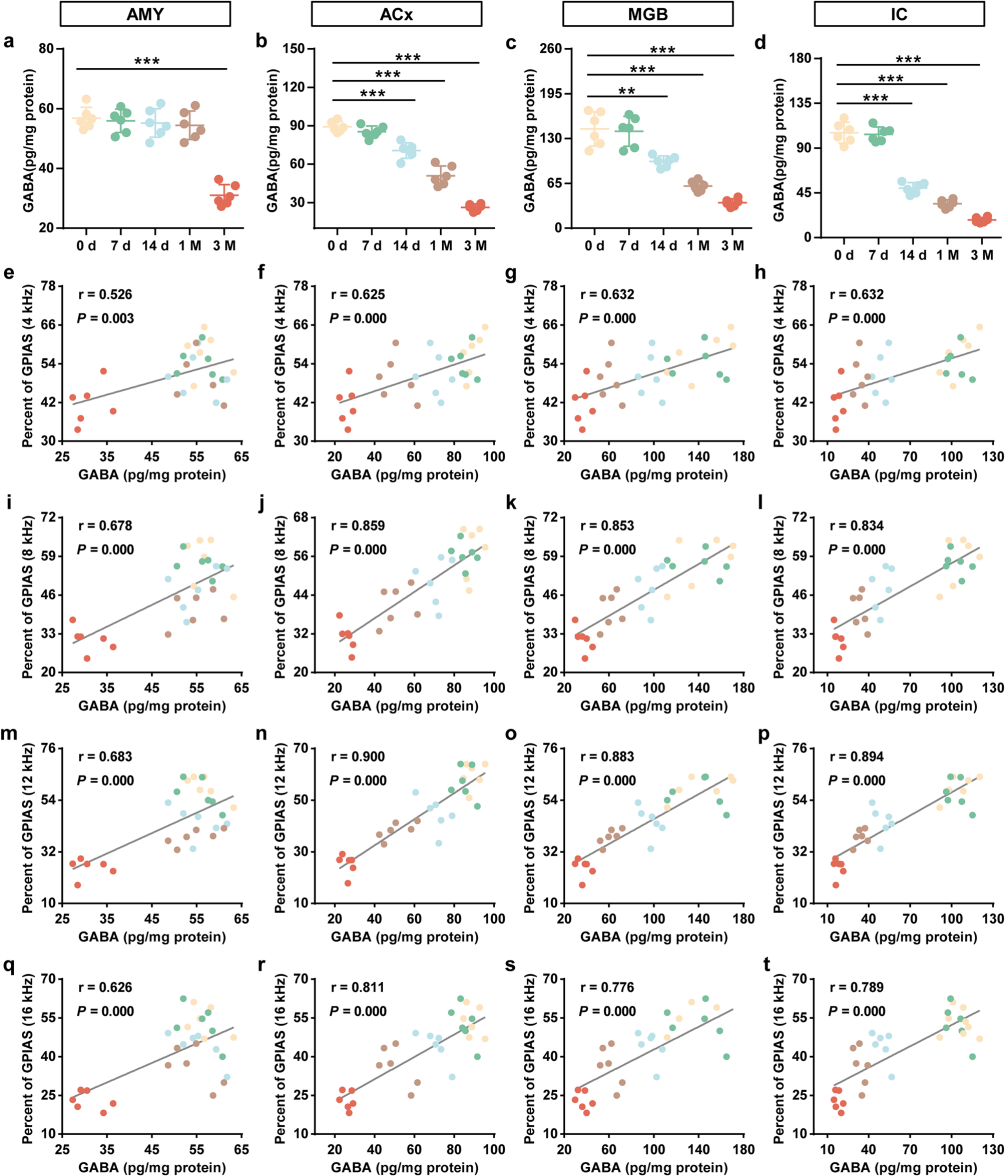
The abundance of GABA in rats with cerebral venous congestion and the correlation between GABA concentration in the auditory-associated areas and tinnitus-like behavioral manifestations. **a-d** The contents of GABA in the AMY (**a**), ACx (**b**), MGB (**c**), and IC (**d**) regions at 0 d, 7 d, 14 d, 1 M, and 3 M after JVL (*n* = 6). **e-t** The correlation between GABA concentration and GPIAS values at 4, 8, 12, and 16 kHz in the AMY (**e, i, m, q**), ACx (**f, j, n, r**), MGB (**g, k, o, s**), and IC (**h, l, p, t**). All data are presented as the mean ± SD. Statistical significance was calculated using a two-tailed unpaired Student’s t test (**a-d**) or Spearman’s rank correlation test (**e-t**). Dots depict individual samples. **p < 0.01; ***p < 0.001 compared to the 0 d group

GABA, the pivotal inhibitory neurotransmitter, participates in the physiological transmission of acoustic information and in hyperacusis [26]. Our previous studies have found lower GABA levels in the cerebrospinal fluid of cerebral venous congestion patients. Thus, we first measured the amount of GABA (including GABA in the vesicle and synaptic cleft) in different brain regions and found GABA concentration significantly decreased in the AMY/ACx/MGB/IC 3 months postoperatively (Fig. 4a-d). Considering the profound effect of GABA on CNS metabolic activity and neuroplasticity [27], we then evaluated the effects of GABA deficiency on cerebral venous congestion-evoked tinnitus-like behavioral phenotypes. The results of correlation analyses showed that the contents of GABA in the cerebral auditory-associated areas were positively correlated with the GPIAS values at 4 kHz (*r* = 0.526, *P* = 0.003 for AMY; *r* = 0.625, *P* = 0.000 for ACx; *r* = 0.632, *P* = 0.000 for MGB; *r* = 0.632, *P* = 0.000 for IC; Fig. 4e-h), 8 kHz (*r* = 0.678, *P* = 0.000 for AMY; *r* = 0.859, *P* = 0.000 for ACx; *r* = 0.853, *P* = 0.000 for MGB; *r* = 0.834, *P* = 0.000 for IC; Fig. 4i-l), 12 kHz (*r* = 0.683, *P* = 0.000 for AMY; *r* = 0.900, *P* = 0.000 for ACx; *r* = 0.883, *P* = 0.000 for MGB; *r* = 0.894, *P* = 0.000 for IC; Fig. 4m-p), and 16 kHz (*r* = 0.626, *P* = 0.000 for AMY; *r* = 0.811, *P* = 0.000 for ACx; *r* = 0.776, *P* = 0.000 for MGB; *r* = 0.789, *P* = 0.000 for IC; Fig. 4q-t). Meanwhile, we found GABA deficiency in the PFC, HP, and CRB (Supplementary Fig. 3a-c) of cerebral venous congestion rats, along with a positive correlation between GABA concentration in the above brain regions and tinnitus-like behavioral phenotypes (Supplementary Fig. 3d-o). Overall, these findings imply that GABA exhibits the ability to transduce neural excitatory signals into cerebral venous congestion-evoked tinnitus.

### Cerebral venous congestion provokes tinnitus by upregulating neuroplasticity-related proteins

**Fig. 5:**
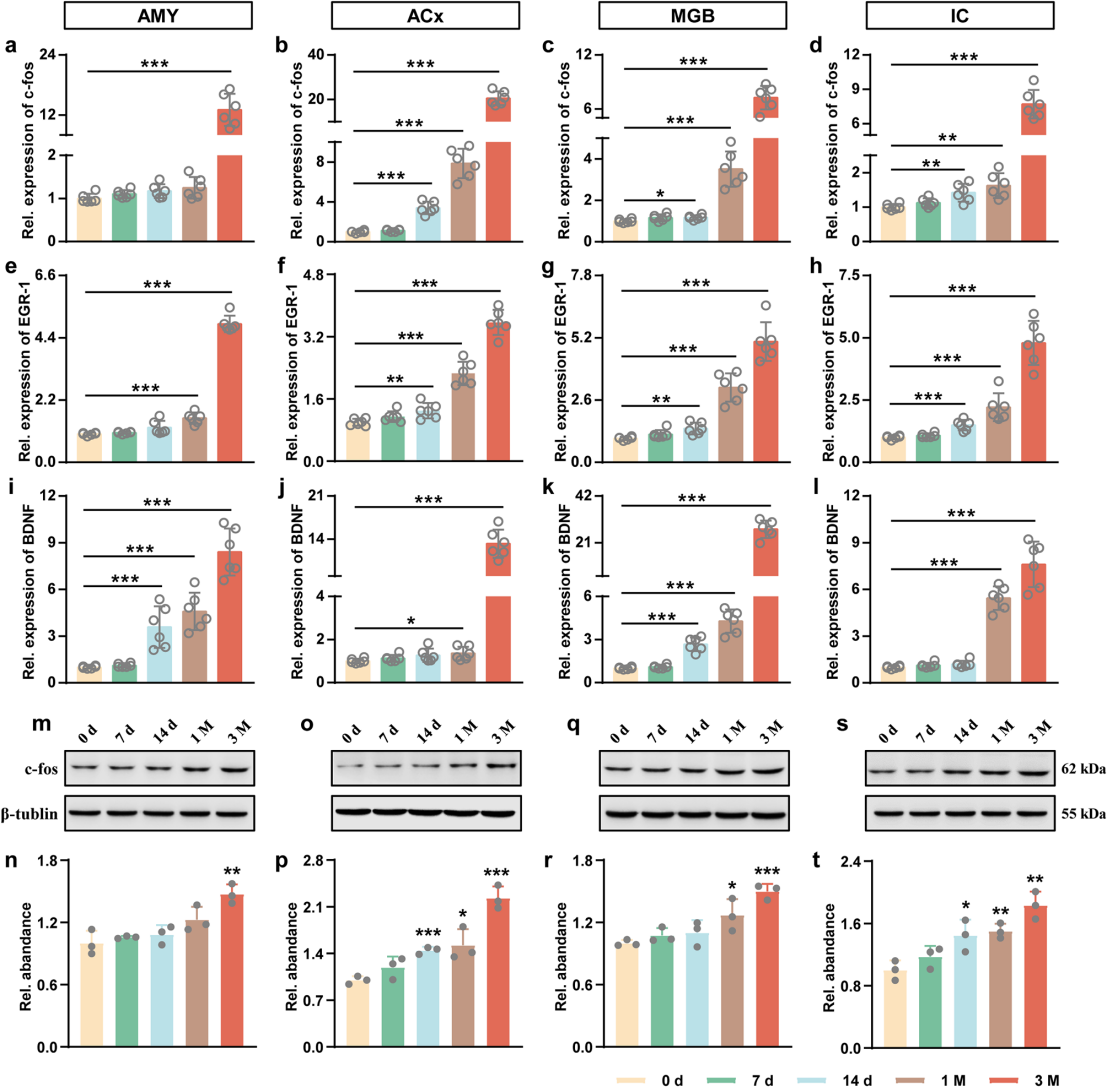
The influence of cerebral venous congestion on neuroplasticity-related proteins in the auditory-associated areas. **a-l** The mRNA expression of c-fos (**a-d**), EGR-1 (**e-h**), and BDNF (**i-l**) in the AMY, ACx, MGB, and IC regions at 0 d, 7 d, 14 d, 1 M, and 3 M after JVL (*n* = 6). **m-t** The protein expression of c-fos in the AMY (**m, n**), ACx (**o, p**), MGB (**q, r**), and IC (**s, t**) at 0 d, 7 d, 14 d, 1 M, and 3 M after JVL (*n* = 3). All data are presented as the mean ± SD (two-tailed unpaired Student’s t test). Dots decipt individual samples. ns, not significance; *p < 0.05; **p < 0.01; ***p < 0.001

**Fig. 6:**
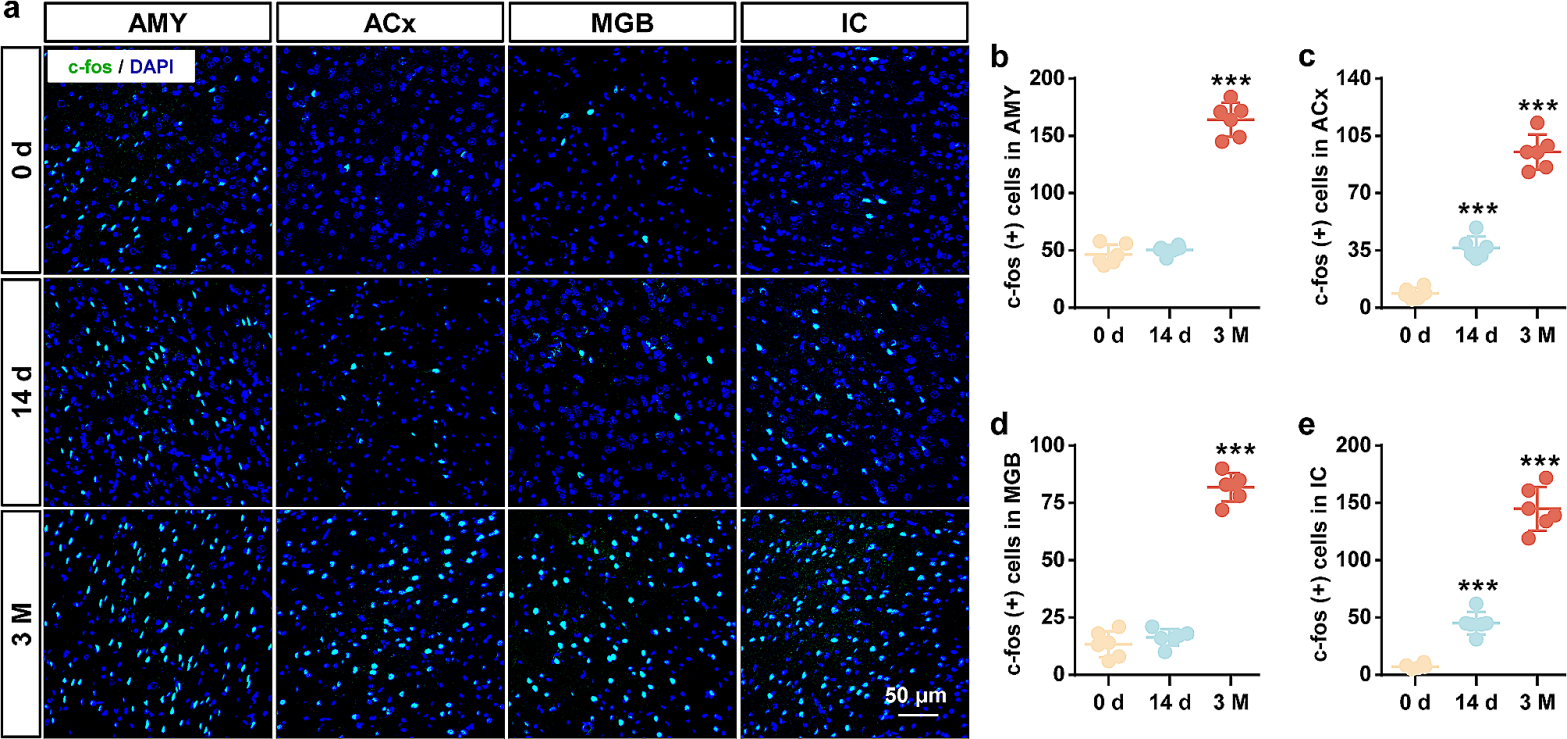
Cerebral venous congestion accelerated the expression of c-fos in the auditory-associated areas. **a** Representative immunofluorescence images of c-fos (green) in the AMY, ACx, MGB, and IC regions at 0 d, 14 d, and 3 M after JVL. **b-e** Quantification of c-fos (+) cells in the AMY (**b**), ACx (**c**), MGB (**d**), and IC (**e**). Scale bar = 50 μm. All data are shown as the mean ± SD (two-tailed unpaired Student’s t test) and are representative of at least five independent experiments. Dots depict individual samples. ***p < 0.001 compared to the 0 d group

**Fig. 7:**
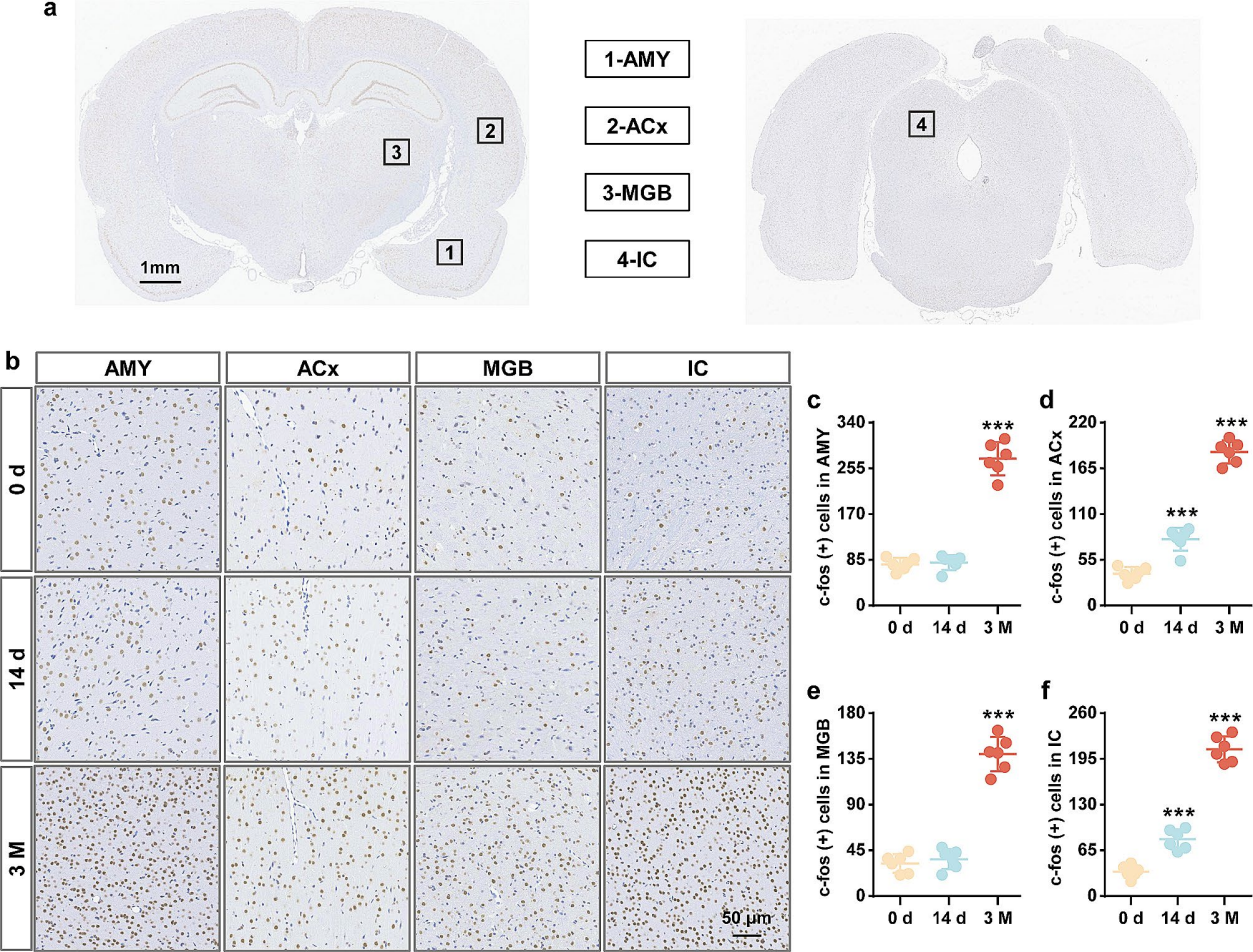
Cerebral venous congestion improved the number of c-fos-positive cells in the auditory-associated areas. **a-b** Representative immunohistochemical images of c-fos in whole-brain sections (**a**) as well as the AMY, ACx, MGB, and IC regions (**b**). **c-f** Quantification of c-fos-positive cells in the AMY (**c**), ACx (**d**), MGB (**e**), and IC (**f**) at 0 d, 14 d, and 3 M after JVL. Scale bar = 50 μm. All data are shown as the mean ± SD (two-tailed unpaired Student’s t test) and are representative of at least five independent experiments. Dots depict individual samples. ***p < 0.001 compared to the 0 d group

To identify the neural mechanisms underlying cerebral venous congestion-evoked tinnitus, we detected synapse-related genes in auditory-associated areas. The qPCR results showed that the mRNA levels of c-fos, EGR-1, and BDNF in the 3 M group were significantly higher than those in the 0 d group (Fig. 5a-l; Supplementary Fig. 4a-i). Since c-fos is known as a validated indirect marker of neuronal activation, we next measured c-fos protein levels. The expression of c-fos showed a tendency to increase in the 3 M group compared to the 0 d group, as shown via Western blotting (Fig. 5m-t; Supplementary Fig. 4j-o). Furthermore, the results of c-fos immunofluorescence were consistent with the expression of the c-fos protein (Fig. 6). From immunohistochemical analysis, the number of c-fos-positive cells was also augmented in the AMY, ACx, MGB, and IC (Fig. 7). The above results support the notion that cerebral venous congestion may regulate tinnitus-like states via c-fos/EGR-1/BDNF.

## Discussion

The results of this study verified that cerebral venous congestion does not contribute to hearing impairment but can evoke tinnitus-like behavior in a validated rat model, as well as illustrated the relevant neurophysiological mechanism. We found that GABA deficiency establishes a direct connection between metabolic abnormalities and neuroplasticity-related inhibitory mechanisms, thereby linking neuronal excitability signaling to cerebral venous congestion-evoked tinnitus-like states.

Cerebrovascular homeostasis depends on continuous blood perfusion to deliver oxygen and glucose in order to maintain a normal brain metabolic state [28, 29]. Because 70% of the cerebral blood volume is located in the veins, the regulation of the cerebral venous system is as vital as that of the cerebral artery system [1, 3, 30, 31]. During natural cerebral circulation, cerebral autoregulation prevents and protects the brain from overflow-induced injury. When confronted with continuous damage, similar to other systems in the organism, the function of the cerebrovascular system becomes decompensated, ultimately leading to increased energy requirements and further dysregulation of neurovascular and neurometabolic coupling function. Our previous studies have revealed that cerebral venous congestion can provoke brain metabolite profile alterations in response to CBF change and cerebral hypoperfusion [16]. Accordingly, we speculate that after transient ischemia and hypoxia in the brain, neurons are capable of making homeostatic changes to sustain their steady state and maintain the normal features of the CNS. In contrast, chronic ischemia and hypoxia, due, for example, to cerebral venous congestion, gradually overwhelm neurons, rendering them prone to metabolic compensatory remodeling and disturbing the balance between excitation and inhibition. Concordantly, our micro-PET imaging showed that rats with cerebral venous congestion exhibit elevated 18 F-FDG uptake in various brain regions in response to the enhanced energy demand of neurons during the cerebral circulation decompensation period. Intriguingly, the dynamic changes in 18 F-FDG uptake are consistent with the fluctuation of tinnitus-like behaviors over time, similar to the previous consensus from studies of salicylate-induced tinnitus [32, 33]. In addition, numerous human PET imaging studies [34, 35] have affirmed that both noise-induced and age-induced tinnitus are strongly linked with increased metabolic activity in the CNS. Our previous clinical studies [10] also found that tinnitus (22/24) was the most common presentation in cerebral venous congestion patients with abnormal brain metabolism. As such, these phenomena may coincide with the clinical findings that patients with incident or early-stage tinnitus had a favorable prognosis, while those with chronic tinnitus showed resistance to various interventions.

Tinnitus is a common symptom of impaired auditory/extra-auditory processing related to cerebral venous congestion. In the “tinnitus-centralization” mechanism, as discussed in many reports [11, 13, 36, 37], tinnitus originates at the cochlear level and develops in the CNS, resulting in compensatory/homeostatic/(mal)adaptive changes in auditory and extra-auditory circuits, while the exact form and localization of these changes may vary among individuals. This has led to the opinion that tinnitus is a complex condition, with auditory, emotional, mnemonic, and attention control networks (including AMY, ACx, MGB, IC, PFC, HP, and CRB) mediating its generation, maintenance, and severity [38–43]. Increasingly many electrophysiological and neuroimaging studies [42, 44, 45] have focused on changes related to central auditory homeostatic plasticity, especially neural hyperexcitability (e.g., enhanced neural spontaneous firing rates) and inhibitory mechanisms (e.g., decreased inhibitory synaptic signaling). In this context, we detected central auditory/nonauditory neuroplasticity-mediated alterations (including long-term changes in synaptic ultrastructure and short-term changes in the strength and efficiency of neurotransmission). Under TEM, we observed an increased number of presynaptic vesicles, with greater PSD thickness and lengthened synaptic active zones in the cerebral venous congestion groups. These adaptive changes in synaptic endings reflect elevated speed and efficacy in chemical synaptic transmission, which contributes significantly to neural excitability. Moreover, the reduced synaptic inhibition owing to cerebral venous congestion-induced GABA deficiency in the central auditory/extra-auditory pathways further reinforces the condition. Taken together, aberrant increases in neuronal circuit excitability and decreases in inhibitory GABAergic neurotransmission facilitate tinnitus-related auditory and extra-auditory pathology, linking CNS homeostatic plasticity deficits to cerebral venous congestion-evoked tinnitus-like states. These findings suggest that the tinnitus-like state related to cerebral venous congestion is a consequence of partially overlapping, dynamically changing, interacting networks. The interaction of these networks determines the phenomenology of tinnitus, ultimately leading to a dimensional disorder that varies in severity of the symptoms and complexity of neural networks.

Some immediate-early genes (IEGs), especially c-fos and EGR-1, are considered to be highly associated with neural activity, synaptic efficacy, and related plasticity changes in neurons [46, 47]. As an essential neurotrophin, BDNF has been reported to play a prominent role during plasticity-associated changes in synaptic efficacy [48–50]. In this study, we found that the expression of c-fos, EGR-1, and BDNF genes was significantly increased within the central auditory system in rats with cerebral venous congestion. Likewise, our immunoblotting and immunostaining data revealed elevated c-fos protein levels. Moreover, the upregulation of these genes is closely correlated with behavioral manifestations, metabolic activity, and synaptic ultrastructural changes. The results of these alterations indicate that c-fos, EGR-1, and BDNF are probably involved in the regulation of neural excitability in response to cerebral venous congestion-related tinnitus. Accordingly, further study is necessary to validate and characterize the neurosynaptic, intracellular, and pericellular environment in a state of cerebral venous congestion-related tinnitus to better understand the molecular crosstalk underlying these correlations.

## Conclusion

In summary, our study demonstrated that rats with cerebral venous congestion developed tinnitus-like behavioral manifestations 14 days postoperatively, after which they had persistent tinnitus without significant hearing impairment. Neuroimaging and neurochemical findings provide strong evidence illustrating that impaired cerebral circulation, increased brain metabolic activity, upregulated synaptic efficacy, and reduced inhibitory synaptic transmission (GABA) are early items in the cascade of pathophysiological events leading to cerebral venous congestion-induced tinnitus. These findings will help to further reveal the neurophysiologic mechanism of tinnitus related to cerebral venous congestion and support the development of precise interventions to interrupt its pathogenesis.

### Electronic supplementary material

Below is the link to the electronic supplementary material.

Supplementary Material 1

Supplementary Material 2

Supplementary Material 3

## Data Availability

The data supporting the findings of this study are included in the supplemental material. Additional data are available from the corresponding author upon reasonable request.

## Abbreviations

CVS: cerebral venous system
CNS: central nervous system
BBB: blood-brain barrier
CBF: cerebral blood flow
JVL: jugular vein ligation
ABR: auditory brainstem responses
SPL: sound pressure level
GPIAS: gap prepulse inhibition of acoustic startle
18F-FDG: 18 F-fluorodeoxyglucose
CT: computed tomography
TEM: Transmission electron microscopy
AMY: amygdala
AC: auditory cortex
MGB: medial geniculate body
IC: inferior colliculus
PFC: prefrontal cortex
HP: hippocampus
CRB: cerebellum
PSD: postsynaptic density
EGR-1: early growth response gene-1
BDNF: brain-derived neurotrophic factor
IEGs: immediate-early genes
