# Supplementary material for: Cerebral venous congestion alters CNS homeostatic plasticity, evoking tinnitus-like behavior
Source: Cell Biosci. 2024 Apr 9;14:47. doi: 10.1186/s13578-024-01221-9 (PMC11003147; doi:10.1186/s13578-024-01221-9)
Supplement: Supplementary file 1 — Supplementary Material 1 [file 13578_2024_1221_MOESM1_ESM.docx]

**Beihang University**

**School of Biological Science and Medical Engineering**

No. 37 Xueyuan Road, Haidian District, Beijing 100191

The People’s Republic of China

Tel: +86-10-83198952

Fax: +86-10-83154745

[jixm@ccmu.edu.cn](mailto:jixm@ccmu.edu.cn)

Beijing, December 30^th^, 2023

Dear Editorial Board of Cell & Bioscience:

Please find enclosed the manuscript: Cerebral venous congestion alters CNS homeostatic plasticity, evoking tinnitus-like behavior (Manuscript ID: CBIO-D-23-00991R1), by Huimin Wei *et al.*, to be submitted as an Original Research Article to Cell & Bioscience. All co-authors have seen and agreed with the contents of the revised manuscript and there are no conflicts of interest to report. We greatly appreciate the insights and questions that the editors and reviewers provided. We have carefully considered all comments from the reviewers and revised our manuscript accordingly. We do hope that our work has the chance to be published by Cell & Bioscience.

Significance of this study: Brain function and neuronal activity depend on a constant supply of blood from the cerebral circulation. The cerebral venous system (CVS) contains approximately 70% of the total cerebral blood volume and plays a critical role in the maintenance of central nervous system (CNS) homeostasis. Similar to the cerebral arterial system, the CVS has vascular regulatory functions (especially neurovascular and neurometabolic coupling) to ensure adequate spatially and temporally targeted delivery of energy substrates in accordance with neuronal activity. Impaired venous autoregulation, which can appear in forms such as cerebral venous congestion, may lead to metabolic abnormalities in the brain, causing severe cerebral functional defects and even chronic tinnitus. As the most frequent complication of impaired auditory/extra-auditory processing related to cerebral venous congestion, tinnitus has a tremendous impact on patients’ psychological and physical health, leading to concentration difficulties, work hindrance, increased prevalence of depression and anxiety, and cognitive decline. Markey *et al.* reported that tinnitus is a representative symptom among cerebral venous congestion patients with idiopathic intracranial hypertension, with an incidence ranging from 52% to 60%. Our previous clinical study found that long-term, continuous tinnitus was present in 60.5% of cerebral venous congestion patients. However, the role of cerebral venous congestion in the progression of tinnitus is underrecognized, and its pathophysiology is still incompletely understood. In this study, we elucidated the specific pathogenetic role of cerebral venous congestion in the onset and persistence of tinnitus and the possible neurophysiological mechanisms underlying CNS homeostatic plasticity malfunction. First, we found that cerebral venous congestion rats exhibited tinnitus-like behavioral manifestations at 14 days postoperatively; from that point onward, they showed signs of persistent tinnitus without significant hearing impairment. Second, neuroimaging and neurochemical findings showed CNS homeostatic plasticity disturbance in rats with cerebral venous congestion, reflected in increased neural metabolic activity, ultrastructural synaptic changes, upregulated synaptic efficacy, reduced inhibitory synaptic transmission (due to GABA deficiency), and elevated expression of neuroplasticity-related proteins in central auditory and extra-auditory pathways. Importantly, we found that GABA deficiency establishes a direct connection between metabolic abnormalities and neuroplasticity-related inhibitory mechanisms, thereby linking neuronal hyperexcitability signaling to cerebral venous congestion-evoked tinnitus-like states. These findings provide a reference for understanding the pathological mechanism of cerebral venous congestion-evoked tinnitus and a new clue for the potential blockade of its development.

We believe that our manuscript fits nicely with Cell & Bioscience’s aim of publishing works on advances in answering important questions relevant to “pathology and mechanisms of neurological disease”, and the readers of Cell & Bioscience will find this paper particularly valuable because our findings may have some implications for the prevention and treatment of neurological disease related to impaired cerebral circulation and may provide valuable information on the management of patients who suffer from cerebral venous congestion. If you have any questions about the manuscript, please let me know.

Thank you very much and I look forward to your reply.

Best regards,

Xunming Ji on behalf of the authors
