# Supplementary material for: Cerebral venous congestion alters CNS homeostatic plasticity, evoking tinnitus-like behavior
Source: Cell Biosci. 2024 Apr 9;14:47. doi: 10.1186/s13578-024-01221-9 (PMC11003147; doi:10.1186/s13578-024-01221-9)
Supplement: Supplementary file 2 — Supplementary Material 2 [file 13578_2024_1221_MOESM2_ESM.docx]

**SUPPLEMENTARY DATA**

**Cerebral venous congestion alters** **CNS homeostatic plasticity,****evoking** **tinnitus-likebehavior**

Huimin Wei^1,2^, Huimin Jiang^2^, Yifan Zhou^2^, Lu Liu^3^, Wei Ma^2^, Shanshan Ni^2,4*^, Chen Zhou^2*^,Xunming Ji^1,2,5*^

^1^Beijing Advanced Innovation Center for Big Data-Based Precision Medicine, School of Biological Science and Medical Engineering, Beihang University, Beijing, 100191, China.

^2^Laboratory of Brain Disorders, Ministry of Science and Technology, Collaborative Innovation Center for Brain Disorders, Beijing Institute of Brain Disorders, Beijing Advanced Innovation Center for Big Data-based Precision Medicine, Capital Medical University, Beijing, 100069, China.

^3^Department of Neurology, Xuanwu Hospital, Capital Medical University, Beijing, 100053, China.

^4^Department of Neurology, Wuqing Hospital of Traditional Chinese Medicine Affiliated to Tianjin University of Traditional Chinese Medicine, Tianjin, 301700, China.

^5^Department of Neurosurgery, Xuanwu Hospital, Capital Medical University, Beijing, 100053, China.

***Corresponding Author:**Xunming Ji, Chen Zhou and Shanshan Ni

Xunming Ji, MD, PhD

E-mail: [jixm@ccmu.edu.cn](mailto:jixm@ccmu.edu.cn)

Address: No. 37 Xueyuan Road, Haidian District, Beijing, 100191, China

Tel.: +86-10-83198952

Chen Zhou, MD, PhD

E-mail: [chenzhou2013abc@163.com](mailto:chenzhou2013abc@163.com)

Address: No.10 Xitoutiao, You An Men, Beijing 100069, China

Tel.: +86-10-83198952

Shanshan Ni, MD

E-mail: nishanshan521@126.com

Address: No.10 Xitoutiao, You An Men, Beijing 100069, China

Tel.: +86-10-83198952


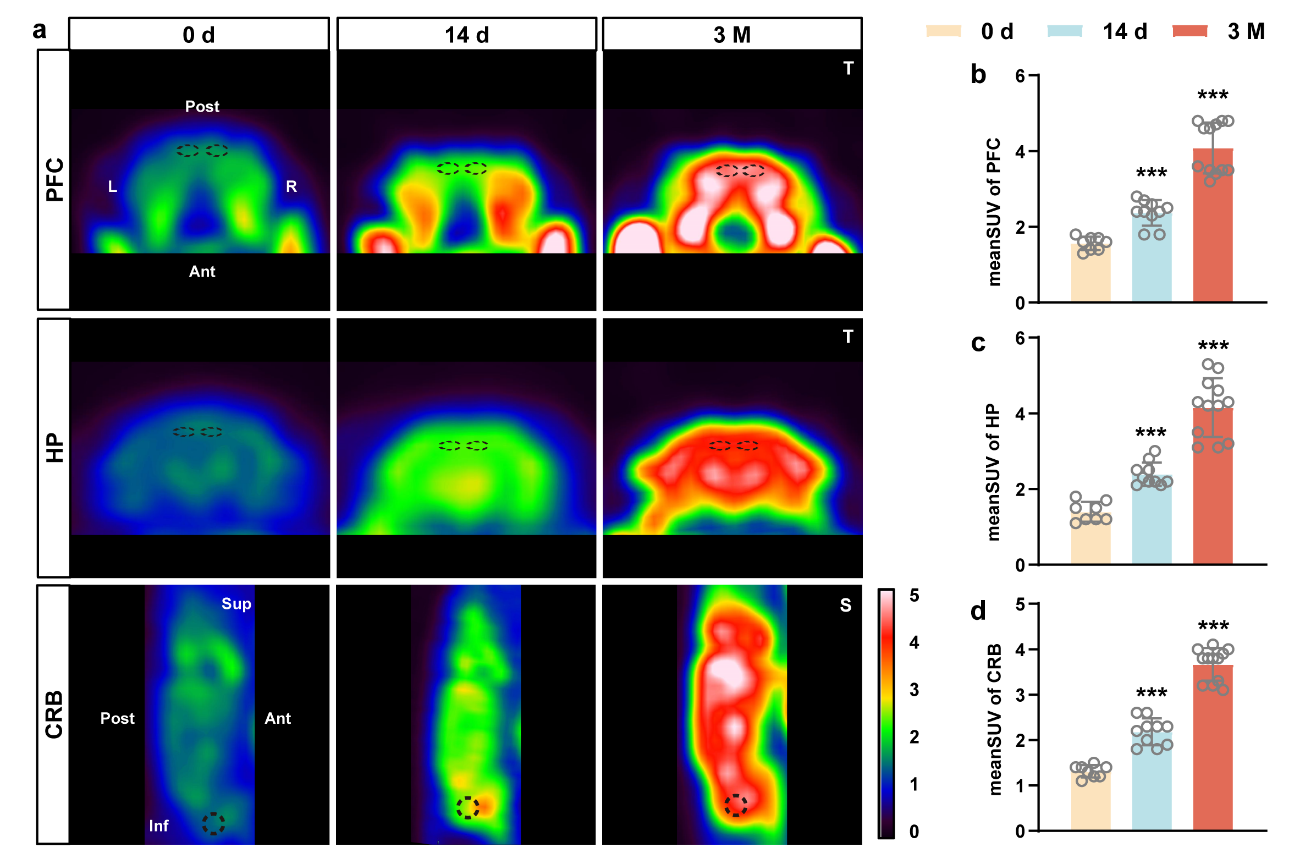


**Supplementary Fig. 1.** Neural metabolic activity was increased in the nonauditory areas of rats with cerebral venous congestion. **a** Representative transaxial or sagittal microPET images of JVL rats at 0 d/14 d/3 M postoperatively. Dotted areas identify PFC, HP, and CRB regions at the corresponding level. PET images are displayed according to an identical color scale, values ranging from 0 to 5 indicate the elevation of 18F-FDG uptake. All rats were imaged in the prone position. Post, posterior; Ant, anterior; Sup, superior; Inf, inferior; L, left; R, right; T: transaxial; S, sagittal. **b-d** Standard uptake value ratio of 18F-FDG in the PFC (**b**), HP (**c**), and CRB(**d**). All data are presented as the mean ± SD (two-tailed unpaired Student’s ttest). Data are representative of at least five independent experiments. Dots depict individual samples.***p < 0.001 compared to the 0 d group.


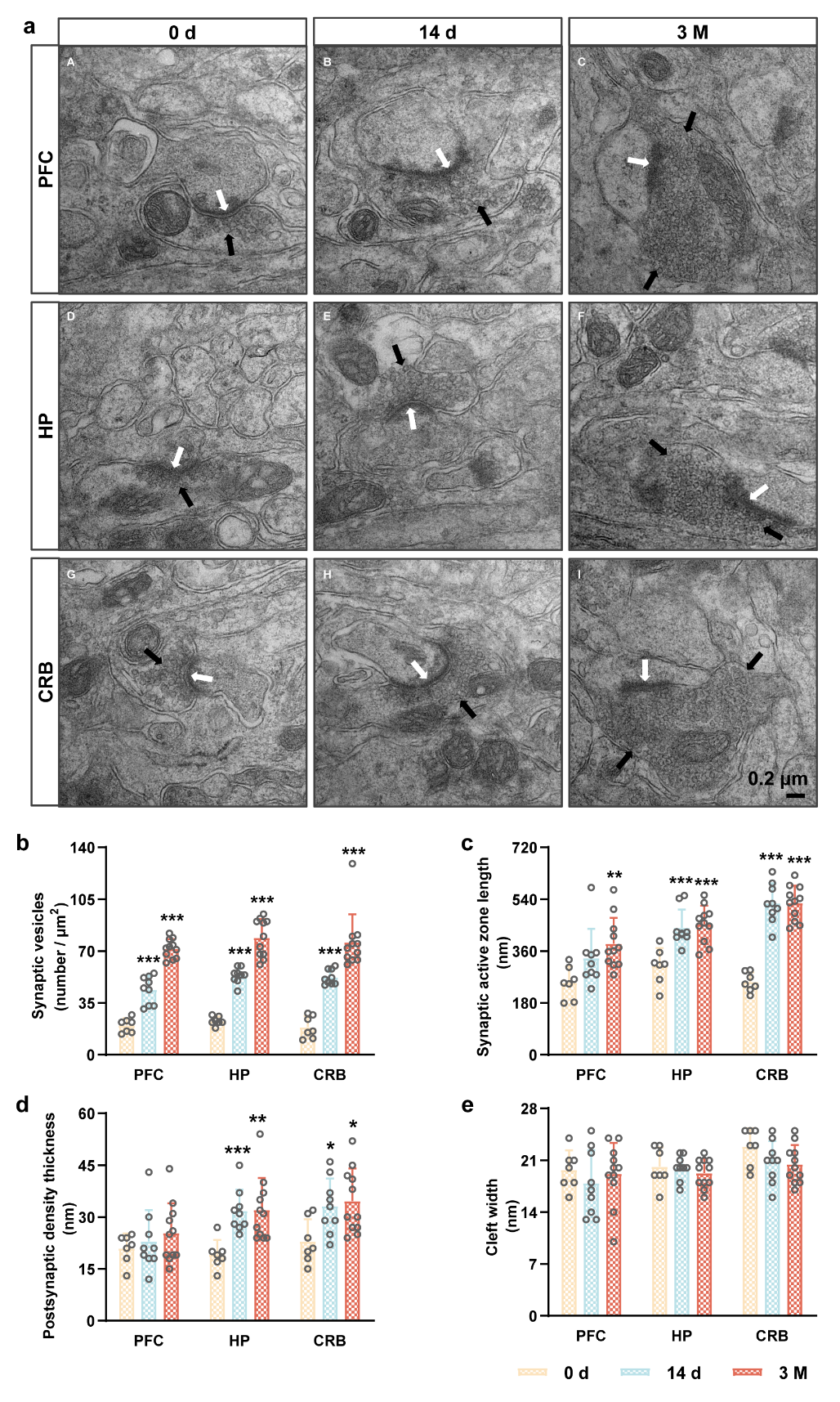


**Supplementary Fig. 2.**Cerebral venous congestion induced ultrastructural changes in synapses in the nonauditory areas.**a** Representative electron micrograph of PFC, HP, and CRB sections in JVL rats at 0 d/14 d/3 M postoperatively. White arrowheads indicate PSD, and black arrowheads indicate presynaptic vesicles. Scale bar = 0.2 μm. **b-e**The number of synaptic vesicles (**b**), length of the synaptic active zone (**c**), PSD thickness (**d**), and synaptic cleft width (**e**) in the PFC, HP, and CRB. All data are presented as the mean ± SD (two-tailed unpaired Student’s ttest) and are representative of at least five independent experiments. Dots depict individual samples.ns, not significance; *p < 0.05; **p < 0.01; ***p < 0.001.


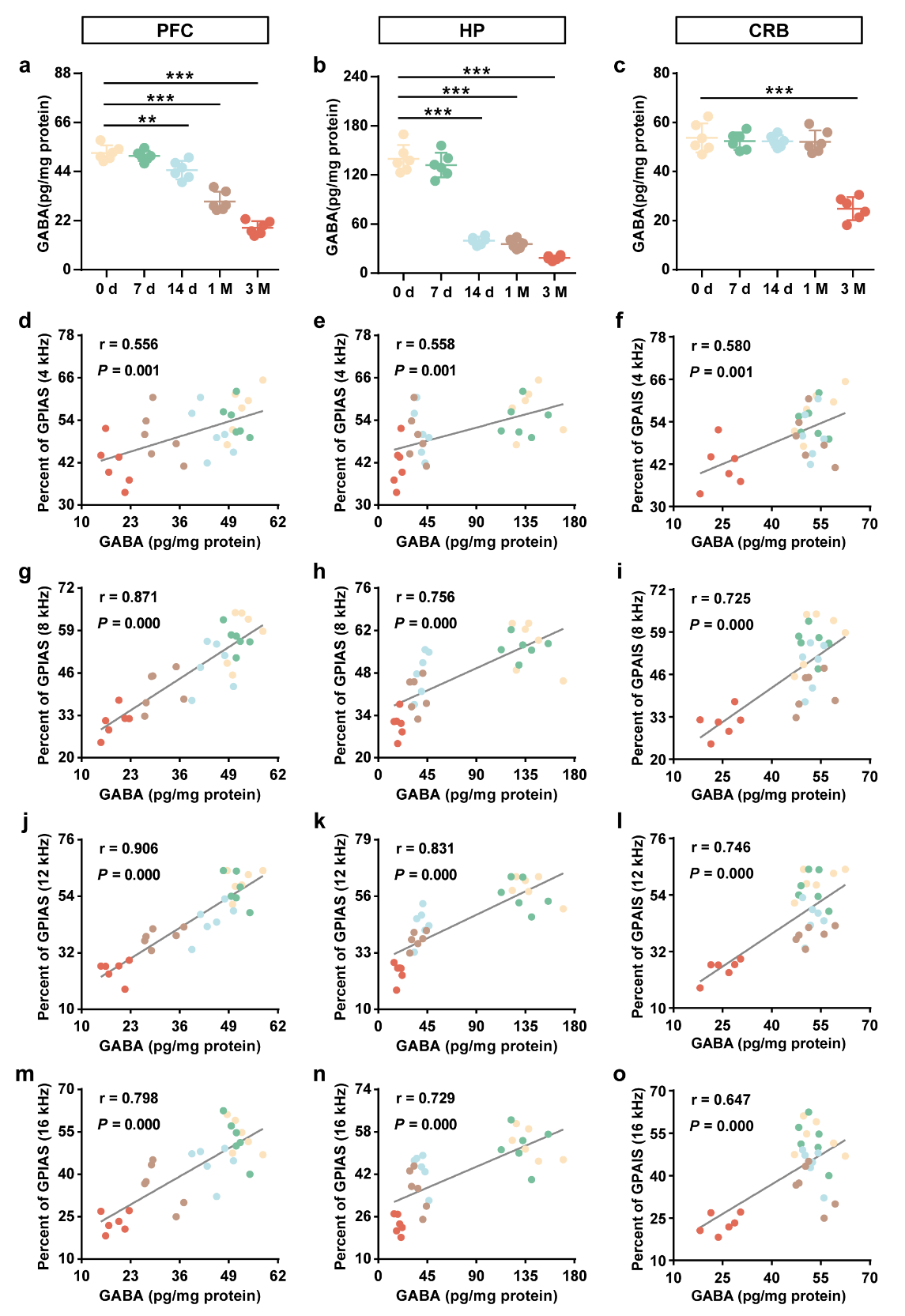


**Supplementary Fig. 3.**The abundance of GABA in rats with cerebral venous congestion and the correlation between GABA concentration in the nonauditory areas and tinnitus-like behavioral manifestations.**a-c**The contents of GABA in the PFC (**a**), HP (**b**), and CRB (**c**) regions at 0 d, 7 d, 14 d, 1 M, and 3 M after JVL (n = 6). **d-o**The correlation between GABA concentration and GPIAS values at 4, 8, 12, and 16 kHz in the PFC (**d, g, j, m**), HP (**e, h, k, n**), and CRB (**f, i, l, o**). All data are presented as the mean ± SD. Statistical significance was calculated using a two-tailed unpaired Student’s ttest (**a-c**) or Spearman’s rank correlation test (**d-o**).Dots depict individual samples. **p < 0.01; ***p < 0.001 compared to the 0 d group.


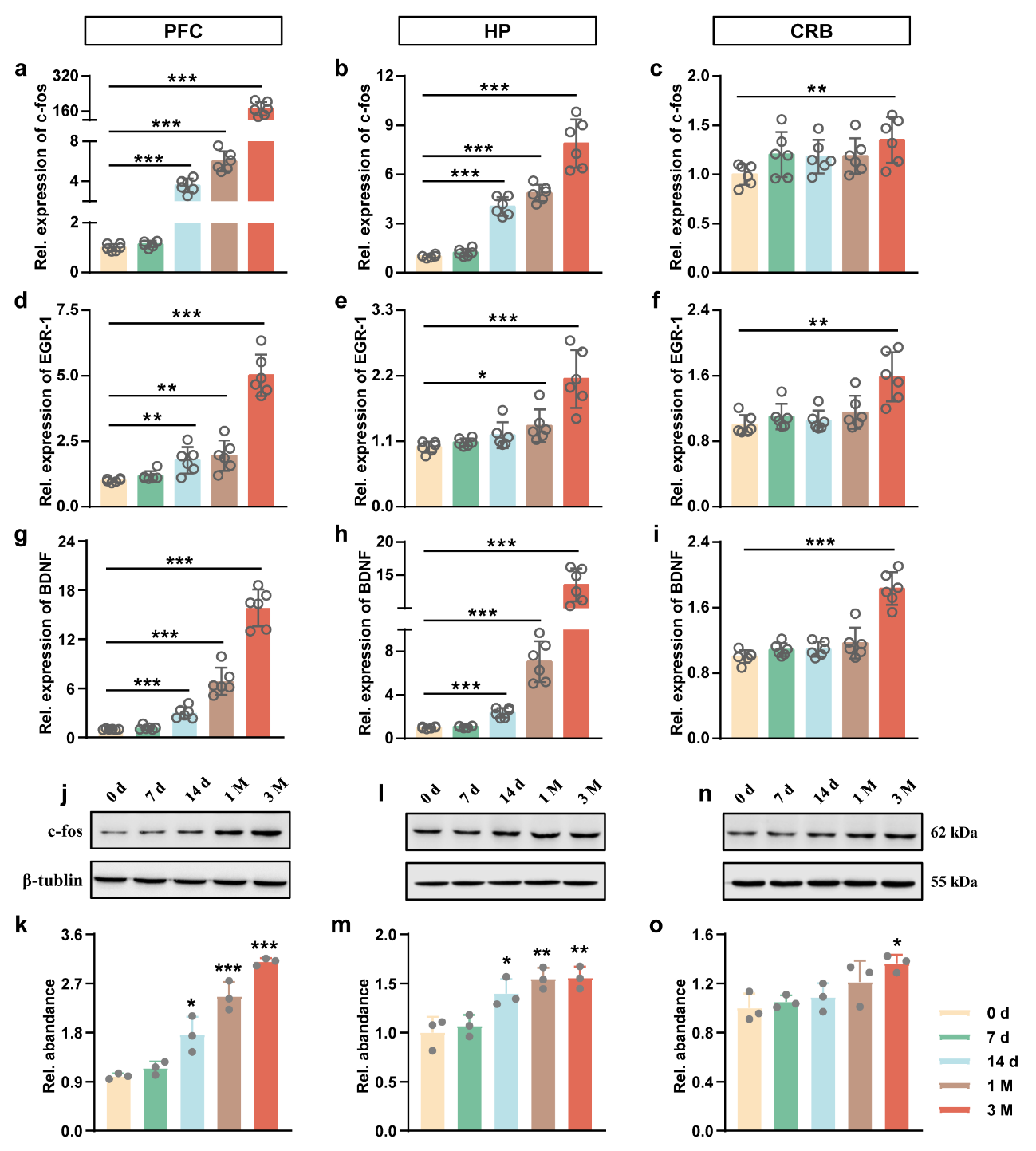


**Supplementary Fig. 4.**The influence of cerebral venous congestion on neuroplasticity-related proteins in the nonauditory areas. **a-i**The mRNA expression of c-fos (**a-c**), EGR-1 (**d-f**), and BDNF (**g-i**)in the PFC, HP, and CRB regions at 0 d, 7 d, 14 d, 1 M, and 3 M after JVL. **j-o**The protein expression of c-fosin the PFC (**j, k**), HP (**l, m**), and CRB (**n, o**) at 0 d, 7 d, 14 d, 1 M, and 3 M after JVL. All data are presented as the mean ± SD (two-tailed unpaired Student’s ttest). Dots depict individual samples.ns, not significance; *p < 0.05; **p < 0.01; ***p < 0.001.

**Supplementary Table 1. List of primers used in qPCR assay.**

| Primer | Nucleotide sequence 5′-3′ | Amplification size (bp) |
| --- | --- | --- |
| c-fos-Forward | CGGTCAAGAACATTAGCAACAT | 129 |
| c-fos-Reverse | AGGAACCAGACAGGTCCACAT |  |
| EGR-1-Forward | AGACAAGTTATCCCAGCCAAA | 202 |
| EGR-1-Reverse | GCAGAGGAAGACGATGAAGC |  |
| BDNF-Forward | ACAGTATTAGCGAGTGGGTCA | 214 |
| BDNF-Reverse | GATTGGGTAGTTCGGCATT |  |
