# Supplementary material for: Cerebral venous congestion alters CNS homeostatic plasticity, evoking tinnitus-like behavior
Source: Cell Biosci. 2024 Apr 9;14:47. doi: 10.1186/s13578-024-01221-9 (PMC11003147; doi:10.1186/s13578-024-01221-9)
Supplement: Supplementary file 3 — Supplementary Material 3 [file 13578_2024_1221_MOESM3_ESM.docx]

**Beihang University**

**School of Biological Science and Medical Engineering**

No. 37 Xueyuan Road, Haidian District, Beijing 100191

The People’s Republic of China

Tel: +86-10-83198952

Fax: +86-10-83154745

jixm@ccmu.edu.cn

Beijing, December 30^th^, 2023

Dear Editors and Reviewers:

Thank you very much for your kind review of our paper entitled “Cerebral venous congestion alters CNS homeostatic plasticity, evoking tinnitus-like behavior (Manuscript ID: CBIO-D-23-00991R1)”. We greatly appreciate the insights and questions that the editors and reviewers provided. Those comments are all valuable and helpful for revising and improving our manuscript.

We have carefully considered all comments and revised our manuscript accordingly. Amended portions are highlighted in yello in the manuscript. The content has also been double-checked, and the typos and grammar errors we found have been corrected. In the following section, we summarize our responses to each comment from the reviewers. We believe that our responses have well addressed all concerns from the reviewers, and hope that the correction will meet with approval.

We look forward to your review and reply. Thank you very much for the consideration.

Sincerely,

Xunming Ji on behalf of the authors

**Responds to the reviewers' comments**

**Reviewer #1**

**Comments:**

To ensure a thorough review process, authors should consider all reviewer concerns and provide detailed point-to-point responses. This demonstrates an understanding of feedback and a commitment to improving their work. It is important to acknowledge and address every concern with clarity and precision, enhancing research credibility.

**Response:**

We thank the reviewer for reading our paper carefully and giving positive comments and constructive suggestions. To acknowledge and address every concern with clarity and precision, we have carefully considered all comments from the reviewer and revised our manuscript accordingly. Please find the following detailed point-to-point responses to your comments and suggestions.

**Comment 1:**

Please display the individual biological replicates (data points) overlaid on the histograms of the bar graphs (Figure 1F, 2b-e, 3b-e, 5n-t, S1b-d, S2b-e, S4k-o). It is currently challenging to determine the number of biological replicates versus technical replicates used.

**Response:**

We thank the reviewer for pointing out this issue, which is valuable for improving the accuracy of the manuscript. As suggested by the reviewer, we have modified the figures to clearly display the individual biological replicates (data points) overlaid on the histograms of the bar graphs (Figure 1F, 2b-e, 3b-e, 5n-t, S1b-d, and S2b-e).

The revised Figure **1F** is as follows:

The revised Figure **2b-e** is as follows:

The revised Figure **3b-e** is as follows:

The revised Figure **5n-t** is as follows:

The revised Figure **S1b-d** is as follows:

The revised Figure **S2b-e** is as follows:

The revised Figure **S4k-o** is as follows:

**Comment 2:**

Any mechanistic changes should occur before the onset of pathological changes, such as tinnitus-like states. It was observed that most of the brain region-specific changes, including GABA content, mRNA expression of c-fos, EGR-1, and BDNF, as well as protein expression of c-fos, were not significant 7 or even 14 days after the surgical operation. Determining whether these brain region-specific changes are the cause or consequence of tinnitus-like states is challenging. Additionally, similar changes were also detected in extra-auditory-associated areas, such as the prefrontal cortex (PFC), hippocampus (HP), and cerebellum (CRB), suggesting that cerebral venous congestion may lead to widespread changes throughout the entire brain rather than specific brain regions. This complicates the interpretation of the findings. The observed changes in auditory-associated areas may simply be associations rather than causal relationships. These possibilities should be discussed in the analysis.

**Response:**

Thank you for the above comment. We agree with the reviewer’s opinion that cerebral venous congestion may lead to widespread changes throughout the entire brain and the observed changes in auditory-associated areas may simply be associations. As suggested by the reviewer, we have added these possibilities in the discussion parts (highlighted in yellow).

Recently, cerebral venous congestion is gaining increasing attention from clinical researchers due to a series of nonspecific clinical manifestations, such as headache (48.8%), dizziness (32.6%), tinnitus (60.5%), visual impairment (39.5%), anxiety or depression (37.5%), and subjective memory decline (30.2%), which impair the quality of life in affected individuals but cannot be explained by other well-established causes[1, 2]. Compared with impaired arterial circulation, cerebral venous congestion is a heterogeneous pathological process that displays more complex and widespread changes throughout the entire brain. Using a validated cerebral venous congestion rat model by extracranial and internal jugular vein ligation (JVL)[3-5], our previous research found that cerebral venous congestion is a complex matrix of behavioral, biological, and neurophysiological phenotypes[6]. Moreover, cerebral venous congestion can lead to a wide range of neuropathological consequences, including blood-brain barrier (BBB) disruption[4], neuroinflammation[4], exacerbation of neurodegeneration[7], development of cerebral microhemorrhages of venous origin[3], altered production of cerebrospinal fluid[8, 9], impaired function of the lymphatic system[10] and dysregulation of CBF[11].

Tinnitus related to cerebral venous congestion is a common symptom of impaired auditory/extra-auditory processing. In the “tinnitus-centralization” mechanism, as discussed in many reports[12-15], tinnitus originates at the cochlear level and develops in the CNS, resulting in compensatory/ homeostatic/(mal)adaptive changes in auditory and extra-auditory circuits, while the exact form and localization of these changes may vary among individuals. This has led to the opinion that tinnitus is a complex condition, with auditory, emotional, mnemonic, and attention control networks (including AMY, ACx, MGB, IC, PFC, HP, and CRB) mediating its generation, maintenance, and severity[16-22]. As shown in Figure 8[22, 23], the pathway transducing sound to the CNS begins as ***cochlear hair cells*** transmit signals via the auditory nerve to the ipsilateral ***cochlear nucleus*** in the pons. From there, most fibers of the tonotopically arranged lemniscal (or classical auditory) pathway cross the midline to synapse in the superior olivary complex. Neurons then ascend via the lateral lemniscus to synapse in the ***IC*** in the midbrain. The IC relays signals to the ***MGB of the thalamus***, from which neurons project to the ***primary AC*** in Heschl gyrus of the temporal lobe.

**Figure Ⅰ-1** Simplified representation of auditory and nonauditory pathways in tinnitus.

In this study, we found that rats with cerebral venous congestion developed tinnitus-like behavioral manifestations 14 days postoperatively, after which they had persistent tinnitus without significant hearing impairment. Subsequent neuroimaging and neurochemical findings showed CNS homeostatic plasticity disturbance in rats with cerebral venous congestion, reflected in increased neural metabolic activity, ultrastructural synaptic changes, upregulated synaptic efficacy, reduced inhibitory synaptic transmission (due to GABA deficiency), and elevated expression of neuroplasticity-related proteins in central auditory and extra-auditory pathways. The results suggest that the tinnitus-like state related to cerebral venous congestion is a consequence of partially overlapping, dynamically changing, interacting networks. The interaction of these networks determines the phenomenology of tinnitus, ultimately leading to a dimensional disorder that varies in severity of the symptoms and complexity of neural networks.

**Comment 3:**

Please provide information regarding the sampling time for GABA content. In Figure 4a-d and S3a-c, the differently colored dots represent GABA measurements at various sampling times after the surgery. These GABA measurements were used for correlation analysis with GPIAS. Similarly, please apply these color schemes to the remaining Figures 4e-t and S3d-o.

**Response:**

Thank you for pointing out this problem, which is valuable for improving the accuracy of this manuscript. Following your suggestion, we have modified the figures and applied corresponding color schemes regarding the sampling time for GABA content in the correlation analysis (Figure 4e-t and S3d-o).

The revised Figure **4e-t** is as follows:

The revised Figure **S3d-o** is as follows:

**Comment 4:**

When measuring GABA content, it is important to note that this refers to the levels of GABA specifically in brain regions, including GABA in the vesicle and synaptic cleft. However, the obtained value does not accurately reflect GABA transmission, but rather pertains to the overall content of GABA. This measurement provides a better reflection of GABA synthesis.

**Response:**

Thank you very much for this significant comment. As suggested by the reviewer, we have added detailed descriptions of “the amount of GABA (including GABA in the vesicle and synaptic cleft) in different brain regions” on page 10 (lines 6-8, highlighted in yellow) to note the specific localization of GABA we examined. *The revised text is as follows:*

Thus, we first measured the amount of GABA (including GABA in the vesicle and synaptic cleft) in different brain regions and found GABA concentration significantly decreased in the AMY/ACx/MGB/IC 3 months postoperatively.

As reported, GABA tone in the brain is primarily generated and regulated by brain cells, particularly astrocytes and neurons, owing to the impermeability of the blood-brain barrier to GABA[24]. The regulation of GABA tone consists of biosynthesis of GABA in and release of GABA from. Essentially, GABA biosynthesis increases intracellular GABA, which then increases the amount of extracellular GABA through various GABA-releasing mechanisms in each cell[25]. The amount of released extracellular GABA constitutes GABA tone and regulates synaptic transmission by inducing the tonic GABA current[25]. Consequently, GABA synthesis is not merely a major contributor to the regulation of GABA tone but also may be used as an indirect index to reflect GABA transmission. In our study, the overall contents of GABA (including GABA in the vesicle and synaptic cleft) in different brain regions (AMY/ACx/MGB/IC/PFC/HP/CRB) were measured by Elisa. The results demonstrated GABA concentration in these regions of JVL rats (3 months postoperatively) was significantly lower than those in sham rats, reflecting GABA synthesis reduced in these regions. Briefly, the decrease in GABA synthesis indirectly suggests a decrease in its transmission.

**Comment 5:**

Please include a concise description of bilateral extracranial and internal jugular vein ligation (JVL) in the methods section.

**Response:**

Thank you for the above suggestions. We have added a concise description of bilateral extracranial and internal jugular vein ligation (JVL) in the ***Materials and Methods*** section (page 4, lines 7-23, JVL rat model part, highlighted in yellow). The added text is as follows:

***JVL rat model***

JVL model was generated as previously reported[5, 6]. Briefly, rats were deeply anesthetized with 4% isoflurane and subsequently maintained between 1.5-2% isoflurane during the surgical procedure using an isoflurane vaporizer (RWD, Shenzhen, China). Each rat was placed in a dorsally recumbent position under a dissection microscope, and the hair from the neck was removed. A cutaneous midline incision was made on the neck. Blunt dissection of the subcutaneous tissue was performed, and the dorsolateral salivary glands were separated. The internal jugular vein, located in the carotid sheath on both sides (above the internal carotid arteries next to the vagus nerve), was subsequently separated and exposed according to the published protocol of Auletta and co-workers[5], making an oblique incision cross one-third point on the orbital line near the ear, retracting the temporalis muscle dorsally and the exorbital lacrimal gland ventrally, exposing and ligating the retroglenoid vein, which is the extracranial termination of the transverse sinus in rodents. Surgical ligations were performed with 8-0 nonabsorbable surgical sutures. Sham rats received the same skin incision and operation without surgical ligation under isoflurane anesthesia. Once the surgery was completed, the wounds were closed using a 6-0 surgical suture with a simple continuous pattern. Rats were allowed to recover under the heat lamp, and antibiotic ointment was applied over the skin incision, following five consecutive days. All efforts were made to minimize the number of animals used and their suffering.

Once again, thanks so much for your helpful comments and constructive suggestions, which have significantly improved the presentation of our manuscript. We sincerely hope that the correction will meet with approval.

**Reference**

[1] Bai, C., et al., Pathogenesis and Management in Cerebrovenous Outflow Disorders. Aging and Disease, 2021. 12(1): p. 203-222.

[2] Zhou, D., et al., Clinical Characteristics and Neuroimaging Findings in Internal Jugular Venous Outflow Disturbance. Thrombosis and Haemostasis, 2019. 119(2): p. 308-318.

[3] Nyul-Toth, A., et al., Cerebral venous congestion exacerbates cerebral microhemorrhages in mice. GeroScience, 2022. 44(2): p. 805-816.

[4] Fulop, G.A., et al., Cerebral venous congestion promotes blood-brain barrier disruption and neuroinflammation, impairing cognitive function in mice. GeroScience, 2019. 41(5): p. 575-589.

[5] Auletta, L., et al., Original Research: Feasibility and safety of two surgical techniques for the development of an animal model of jugular vein occlusion. Experimental Biology and Medicine (Maywood, N.J.), 2017. 242(1): p. 22-28.

[6] Wei, H., et al., Cerebral venous congestion alters brain metabolite profiles, impairing cognitive function. Journal of Cerebral Blood Flow and Metabolism: Official Journal of the International Society of Cerebral Blood Flow and Metabolism, 2023. 43(11): p. 1857-1872.

[7] Tong, L.S., et al., Cerebral venous collaterals: A new fort for fighting ischemic stroke? Prog Neurobiol, 2018. 163-164: p. 172-193.

[8] Fulop, G.A., et al., Role of age-related alterations of the cerebral venous circulation in the pathogenesis of vascular cognitive impairment. American Journal of Physiology. Heart and Circulatory Physiology, 2019. 316(5): p. H1124-H1140.

[9] Aso, T., et al., A venous mechanism of ventriculomegaly shared between traumatic brain injury and normal ageing. Brain: a Journal of Neurology, 2020. 143(6): p. 1843-1856.

[10] Ghali, M.G.Z., et al., Structure and function of the perivascular fluid compartment and vertebral venous plexus: Illumining a novel theory on mechanisms underlying the pathogenesis of Alzheimer's, cerebral small vessel, and neurodegenerative diseases. Neurobiology of Disease, 2020. 144: p. 105022.

[11] Duncombe, J., et al., Chronic cerebral hypoperfusion: a key mechanism leading to vascular cognitive impairment and dementia. Closing the translational gap between rodent models and human vascular cognitive impairment and dementia. Clinical Science (London, England: 1979), 2017. 131(19): p. 2451-2468.

[12] Henton, A. and T. Tzounopoulos, What's the buzz? The neuroscience and the treatment of tinnitus. Physiol Rev, 2021. 101(4): p. 1609-1632.

[13] Mohan, A., et al., Symptom dimensions to address heterogeneity in tinnitus. Neuroscience and Biobehavioral Reviews, 2022. 134: p. 104542.

[14] Bauer, C.A., Tinnitus. The New England Journal of Medicine, 2018. 378(13): p. 1224-1231.

[15] Noreña, A.J. and B.J. Farley, Tinnitus-related neural activity: Theories of generation, propagation, and centralization. Hearing Research, 2013. 295: p. 161-171.

[16] Leaver, A.M., et al., Dysregulation of limbic and auditory networks in tinnitus. Neuron, 2011. 69(1): p. 33-43.

[17] De Ridder, D., et al., Phantom percepts: tinnitus and pain as persisting aversive memory networks. Proceedings of the National Academy of Sciences of the United States of America, 2011. 108(20): p. 8075-8080.

[18] Roberts, L.E., F.T. Husain, and J.J. Eggermont, Role of attention in the generation and modulation of tinnitus. Neuroscience and Biobehavioral Reviews, 2013. 37(8): p. 1754-1773.

[19] De Ridder, D., et al., Amygdalohippocampal involvement in tinnitus and auditory memory. Acta Oto-laryngologica. Supplementum, 2006(556): p. 50-53.

[20] Chen, Y.-C., et al., Tinnitus and hyperacusis involve hyperactivity and enhanced connectivity in auditory-limbic-arousal-cerebellar network. ELife, 2015. 4: p. e06576.

[21] Adjamian, P., et al., Neuroanatomical abnormalities in chronic tinnitus in the human brain. Neuroscience and Biobehavioral Reviews, 2014. 45: p. 119-133.

[22] Shore, S.E., L.E. Roberts, and B. Langguth, Maladaptive plasticity in tinnitus--triggers, mechanisms and treatment. Nature Reviews. Neurology, 2016. 12(3): p. 150-160.

[23] Adams, M.E., et al., Tinnitus Neuroimaging. Otolaryngologic Clinics of North America, 2020. 53(4): p. 583-603.

[24] Hepsomali, P., et al., Effects of Oral Gamma-Aminobutyric Acid (GABA) Administration on Stress and Sleep in Humans: A Systematic Review. Frontiers In Neuroscience, 2020. 14: p. 923.

[25] Koh, W., et al., GABA tone regulation and its cognitive functions in the brain. Nature Reviews. Neuroscience, 2023. 24(9): p. 523-539.
